# Myoelectric activity along human *gastrocnemius medialis*: Different spatial distributions of postural and electrically elicited surface potentials

**DOI:** 10.1016/j.jelekin.2012.08.003

**Published:** 2013-02

**Authors:** Emma F. Hodson-Tole, Ian D. Loram, Taian M.M. Vieira

**Affiliations:** a Institute for Biomedical Research into Human Movement and Health, Manchester Metropolitan University, Manchester, UK; b Lab. for Eng. of the Neuromuscular System, Dept. of Electronics, Politecnico di Torino, Italy

**Keywords:** Electrode array, Postural control, Surface electromyograms, Electrical stimulation, Motor units, Regional distribution

## Abstract

It has recently been shown that motor units in human medial gastrocnemius (MG), activated during standing, occupy relatively small territories along the muscle’s longitudinal axis. Such organisation provides potential for different motor tasks to produce differing regional patterns of activity. Here, we investigate whether postural control and nerve electrical stimulation produce equal longitudinal activation patterns in MG. Myoelectric activity, at different proximal–distal locations of MG, was recorded using a linear electrode array. To ensure differences in signal amplitude between channels did not result from local, morphological factors two experimental protocols were completed: (i) quiet standing; (ii) electrical stimulation of the tibial nerve. Averaged, rectified values (ARVs) were calculated for each channel in each condition. The distribution of signals along electrode channels was described using linear regression and differences between protocols at each channel determined as the ratio between mean ARV from standing: stimulation protocols. Ratio values changed systematically across electrode channels in seven (of eight) participants, with larger values in distal channels. The distribution of ARV along MG therefore differed between experimental conditions. Compared to fibres of units activated during MG nerve stimulation, units activated during standing may have a tendency to be more highly represented in the distal muscle portion.

## 1 Introduction

During a motor task the number and combination (e.g. different sizes and firing rates) of activated motor units will influence the magnitude of force produced by any one muscle. For many tasks it is rare for all motor units within a muscle to be activated at any one time. It is therefore likely that patterns of activation will vary across the volume of a muscle over the time course of a given task. In muscles with broad attachment sites (e.g. deltoid) this means that anatomical regions within a muscle can be closely tied to functional tasks (English et al., 1993; Brown et al., 2007). However, even within muscles with narrow attachment sites, which may be considered to provide a single line of action, spatially localised patterns of activity can occur in response to different: perturbations (Wolf et al., 1992); mechanical demands (Higham et al., 2008; Wakeling, 2009); force direction demands (Herrmann and Flanders, 1998; Staudenmann et al., 2009) or activation levels (Kinugasa et al., 2011). Although not directly investigated, these examples suggest that functional specialisation may not be limited to muscles with broad attachment sites.

In humans, the medial gastrocnemius (MG) muscle is a plantar flexor of the foot with a simple unipennate fascicle architecture (Wolf and Kim, 1997), which contributes to maintenance of standing posture. Recent evidence has shown that the fibres of MG motor units activated during standing occupy localised territories in the longitudinal plane (∼40 mm, Vieira et al., 2011). As the contribution of MG to postural control is not the result of a stereotyped pattern of behaviour, but appears to result from flexible, selective activation of motor units (Vieira et al., 2010), it is possible that different spatial regions of MG contribute differently to the maintenance of upright standing posture.

Postural control requires low levels of active torque to be produced (<4% MVC ∼4 Nm, Jacono et al., 2004), suggesting that smaller, fatigue resistant units within MG are active during quiet standing. Geometric arrangement of the fibres of these units to provide more direct force transmission to the associated joint(s) could provide a means of economically producing the tension required for postural control. This has been suggested to occur in the cat, where the predominantly slow fibred soleus muscle is composed of long parallel fibres, therefore facilitating more direct force transmission from fibres which generate a greater net impulse (integral of tension × time) (Spector et al., 1980). Fascicle geometries in human MG are pennate in the proximal muscle region (∼38 degrees, Shin et al., 2009), becoming more parallel in the distal region (∼20 degrees, Shin et al., 2009). As the portion of force transmitted to the tendon is a function of the cosine of pennation angle (Lieber and Friden, 2001), fascicles in the distal portion of MG might transmit a greater portion of force produced to the resulting ankle torque than more proximal fibres (based on pennation angles from Shin et al., 2009: ∼94% vs. ∼79%). Fibres in the distal region of the muscle may therefore contribute more economically to torque about the ankle joint. The purpose of this study was therefore to identify whether motor units with fibres activated during the maintenance of standing posture showed a tendency to be more highly represented in the distal portion of MG.

**Fig. 1:**
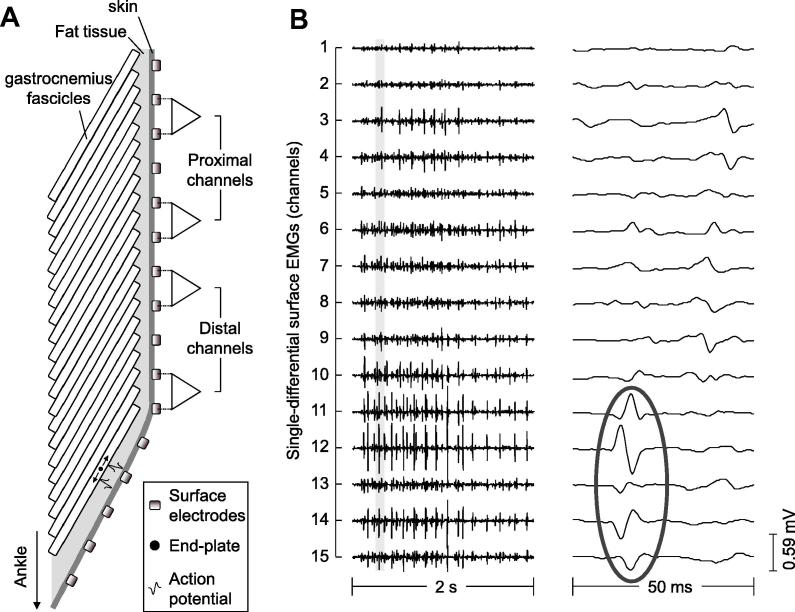
Representation of how the orientation of fascicles within MG influences the patterns of myoelectric activity recorded at different proximal–distal muscle regions. (A) Orientation of fascicles in the proximal muscle region leads to myoelectric activity from different fibres being represented in different channels of the electrode array. Close to the muscle–tendon junction, fascicles are orientated more parallel to the skin, so electrode channels now lie along fascicles and propagation of action potentials will be visible in electrode channels located over the region. (B) Myoelectric activity from one participant recorded during a 2 s period of standing (left panel), with signals from a 50 ms portion of this time (grey block) shown in the right panel. In the right panel signal propagation is visible and highlighted in channels in 11–15, with the motor end plate evident in channel 13.

Due to the pennate fascicle architecture within MG, action potentials travel along the fibres from deep-superficial regions of the muscle, terminating at the superficial aponeurosis. Signals recorded at the skin surface in one region of MG therefore represent activity in fibres attaching close to that location (Mesin et al., 2011). Use of a linear array of electrodes, placed along the proximal–distal length of MG, will therefore provide information on the location of activated fibres along the longitudinal axis of the muscle (Vieira et al., 2011). Here, we use a linear array to record myoelectric signals during standing, quantifying signal amplitude in each channel and revealing the spatial distribution of activated fibres (Fig. 1). To ensure differences in signal amplitude did not merely reflect local factors (e.g. pennation angle, total number of fibres within the detection volume of each electrode etc.), we applied a second experimental protocol to quantify signal amplitude in each electrode channel when a population of motor units, highly unlikely to contribute to postural control, were activated. This was achieved using low-level electrical stimulation of the tibial nerve, which has been shown to largely result in preferential activation of populations of faster motor units (Hennings et al., 2007). Due to the small cross sectional area of the nerve, the current intensity impinging upon the tibial nerve distributes evenly across individual motor axons (Rosenfalck, 1969). For this reason, during nerve stimulation, the activation threshold rather than the axon position within the nerve is the determinant of the electrically elicited motor units. Motor units activated during this protocol were therefore anticipated to be highly unlikely to contribute to postural control during quiet standing.

Using the two experimental protocols we tested the null hypothesis that fibres of motor units activated during standing and electrical stimulation are equally distributed along the proximal–distal length of MG. It was expected that either the amplitude of the myoelectric signal would be similar across electrode channels or that any differences would be systematically related to local geometric or anatomical properties of the muscle, with a similar pattern of distribution occurring whether units were activated during standing or by electrical stimulation. Crucially, if the null hypothesis is true, any systematic patterns of difference would not change when comparing signals from the two experimental protocols.

## 2 Materials and methods

### 2.1 Ethical approval

Experiments were approved by the Academic Ethics Committee of the Faculty of Science and Engineering, Manchester Metropolitan University. Participants gave written, informed consent to the experiments which conformed to the standards set by the latest revision of the *Declaration of Helsinki*.

### 2.2 Data collection

A 16 Ag-bar linear electrode array (10 mm inter-electrode distance) was secured along the length of MG on the left leg of eight participants (seven male). Two experimental protocols were completed. Firstly, we used electrical stimulation of the tibial nerve. Participants were strapped about the waist to a fixed board, enabling them to stand without swaying and with no/minimal activation of the ankle extensor muscles. The electrode array was situated in the position where the strongest M-wave signals were detected when a low level stimulation was applied to the tibial nerve in the region of the popliteal fossa. Compound muscle action potentials were elicited by electrical stimulation (Digitimer DS7, Digitimer Ltd., Welwyn Garden City, UK), applied with the cathode (circular, pre-gelled electrode 10 mm diameter) positioned over the posterior tibial nerve at the level of the popliteal fossa and the anode (80 × 50 mm, damp cloth) fixed to the leg, just above the patella, using elasticised bandage (Vetrap™, 3 M United Kingdom PLC, Bracknell, UK). The stimulation level was set by identifying the lowest amplitude at which a clear M-wave occurred. This amplitude was doubled (mean ± SD: 19.79 ± 5.51 mA), ensuring that clear M-waves occurred through the whole trial and that participants were comfortable and able to maintain a relaxed posture. A 200 μs wide pulse was applied at 1 Hz for 400 s, while myoelectric data (2048 Hz) were recorded.

Following the stimulation protocol myoelectric signals were collected during quiet standing. The waist band and backboard were removed allowing subjects to stand freely on the footplates and myoelectric data were collected over a 40 s trial.

### 2.3 Data analysis

Localised activity, either resulting from the stimulation pulses or during standing, was quantified by calculating the mean amplitude of the surface myoelectric signal recorded by each channel (i.e. pair of electrodes). Initially, myoelectric signals were band-pass filtered (15–350 Hz, 2nd order Butterworth filter). For stimulation data, trigger signals, delivered simultaneously to the stimulation pulses, were used to segment the surface EMGs, providing individual, averaged M-waves represented in epoch of 20 ms. For each channel, M-waves were rectified and, then, averaged. These averaged, rectified values (ARV) reflect the amplitude of M-waves detected across the array of electrodes for each subject:

(1) $$\text{ARV}[\mathrm{ch}]=\frac{1}{41N_{d}}\overset{41}{\underset{i=1}{\sum }}\left|\overset{N_{d}}{\underset{j=1}{\sum }}\mathrm{EMG}[\mathrm{ch}\text{,}i\Delta t+t_{j}]\right|\text{,}$$

where *EMG*[*ch*, *i*Δ*t *+ *t_j_*] stands for the raw surface myoelectric signal recorded with the channel number *ch* (from channel 1 (proximal) to 15 (distal)) from one subject, Δ*t* denotes the sampling interval (1/2048 s) and *i* is an integer number from 1 to 41 inclusively (41 samples equal ∼20 ms). *t_j_* are the instant corresponding to the delivery of the stimulation pulses, with *j* ranging from 1 to the total number of pulses (*N_d_ *= 400). Eq. (1) was used to compute the ARV amplitude for the surface myoelectric signals recorded during the stimulation protocol. During standing, EMGs were not triggered and the ARV amplitude was calculated over the whole recording duration (40 s). Mean ARV values for each channel were calculated for standing from the ARV of 1 s epoch and for stimulation from the ARV of all recorded M-waves.

### 2.4 Quantifying changes in the spatial distribution of myoelectric signals

Central to our methods is the fact that activity in muscle fibres at different proximal–distal locations of MG will influence the myoelectric signal characteristics recorded in different channels along the electrode array (Vieira et al., 2011). The association between the number of active muscle fibres and the local amplitude of the surface myoelectric signal holds only for recordings obtained from locations where the associated fibres do not run parallel to the skin and propagation of action potentials does not occur between adjacent electrode channels (Fig. 1a and see also Vieira et al., 2011). In such instances it is not possible to infer information on the spatial distribution of active fibres across the muscle volume, so channels where action potential propagation occurred were excluded from the analysis.

A comparison of myoelectric signal amplitude between experimental protocols within each channel was determined within each participant by calculating the ratio between mean ARV from each protocol. This effectively normalised each channel to itself whilst still preserving the characteristics of the spatial pattern of signal distributions along the array. The resulting ratios reveal the locations of the largest differences in signal amplitudes between protocols.

### 2.5 Statistical analysis

We wished to identify whether a significant difference in the distribution of myoelectric signal amplitudes across electrode channels occurred between standing and stimulation protocols. To characterise signal amplitude along the electrode array we therefore calculated the line of best fit between electrode channel and mean ARV, using linear regression analysis. We used *T*-test to identify whether the mean difference in slope co-efficient between experimental conditions differed significantly from zero. As a significant linear relationship was not identified in all participants/conditions the test was repeated, replacing the slope co-efficient with zero where no significant linear relationship occurred. Linear regression analysis was also used to quantify distribution of the calculated ratio values across electrode channels. All results were considered significant when *p *⩽ 0.05 and *post hoc* statistical power (1 – probability of falsely retaining the null hypothesis) was calculated using (Faul et al., 2007, 2009).

## 3 Results

**Fig. 2:**
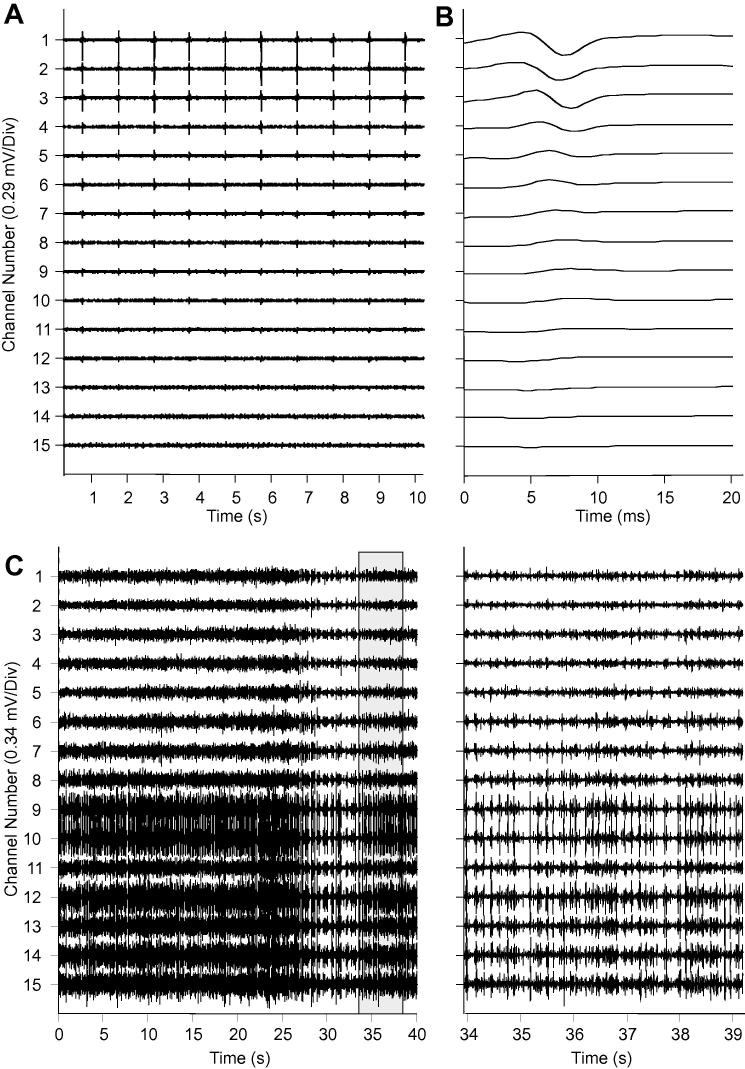
Proximal and distal myoelectric activity in each electrode channel from subject 7 during: (A) stimulation, with the mean M-Wave profiles shown (B); (C) myoelectric activity during standing. The right panel provides a magnified view of a short period within the trial. In A, the stimulation artefact has been removed from the raw signal so all the M-wave profiles are visible.

Fig. 2 provides representative data from one participant. During the stimulation protocol, the largest myoelectric signal amplitudes were localised in the proximal electrode channels, with much smaller/negligible M-waves in the distal channels (Fig. 2a and b). In contrast, during standing the myoelectric signal amplitude was lower in the proximal electrode channels compared to the distal channels (Fig. 2c). These data clearly reveal a change in the distribution of the myoelectric signal amplitude along electrode channels between conditions in this participant.

**Fig. 3:**
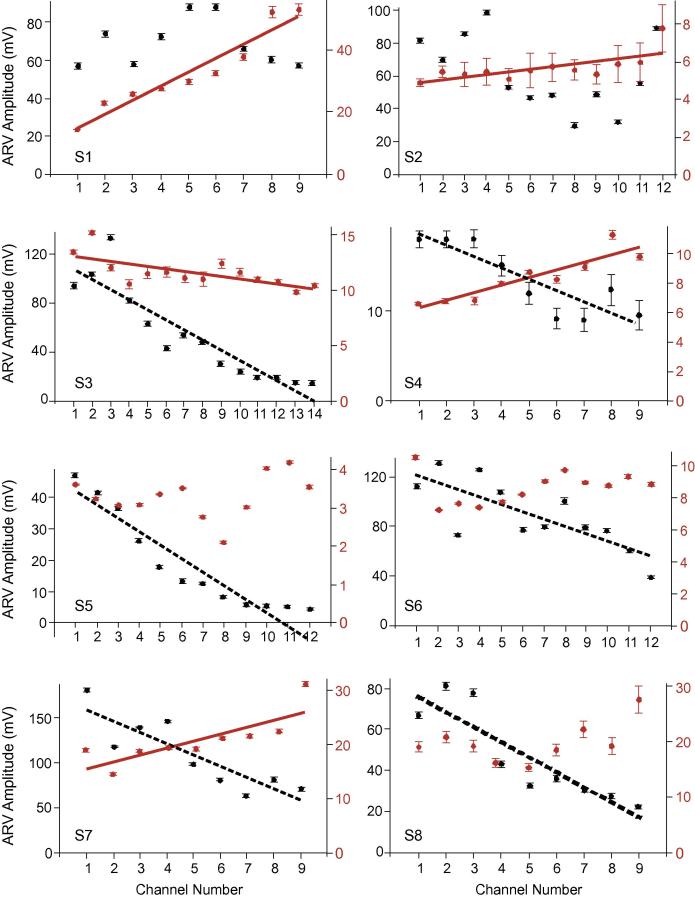
Mean ± S.E.M. ARV across each electrode channel (1 = proximal) from stimulation (black symbols) and standing (red symbols) protocols, for each participant. Where a significant linear relationship exists, the line of best fit is provided. Details of statistical analyses are shown in Table 1. (For interpretation of the references to colour in this figure legend, the reader is referred to the web version of this article.)

Key to our analysis was the distribution of ARV amplitudes across the electrode channels. If the spatial distribution of motor units activated during the two experimental protocols is similar we would expect to see a consistent pattern of variation across the channels in each condition. Fig. 3 shows mean ARV amplitudes across electrode channels for both protocols in each participant, with results of statistical analysis shown in Table 1. In all, except one participant (Fig. 3, see S3), the pattern of ARV amplitudes across electrode channels differed between the two conditions. During standing, regression analysis revealed there was a significantly positive linear relationship between ARV amplitude and electrode channel in five of the eight participants (*p *⩽ 0.01, Fig. 3). During stimulation, regression analysis revealed a significant linear relationship in six of the participants (*p *⩽ 0.003, Fig. 3), with the relationship always negative. The *T*-test, identifying whether mean differences in the slope of the line of best fit between conditions significantly differed from zero, confirmed a significant difference across the group (*p *= 0.003, *N *= 8). When non-significant slope co-efficients were replaced in the data set with zero values, significant differences still existed across the group (*p *= 0.007, *N *= 8). For both tests there was a strong effect size, meaning that even with *N *= 8, statistical power of 0.97 and 0.90 occurred (Faul et al., 2007).

**Fig. 4:**
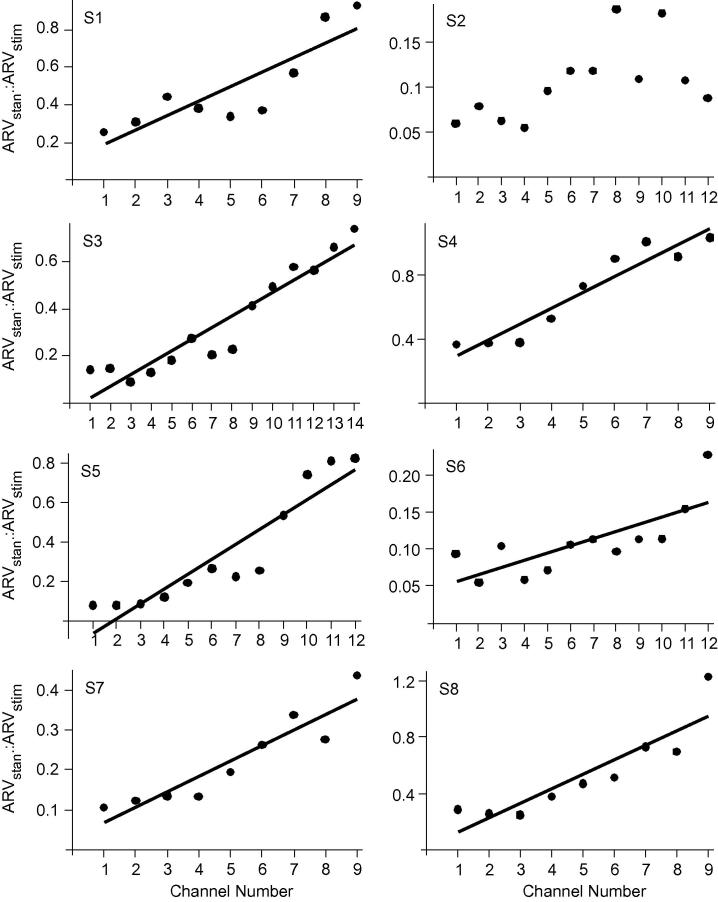
Ratio of ARV values from stimulation and standing protocols for each electrode channel (1 = proximal). Each graph illustrates data from one participant with results of linear regression analysis and line of best fit displayed where a significant relationship occurred. Details of statistical analyses are shown in Table 1.

The ratio between mean ARV’s from stimulation and standing protocols, changed systematically across proximal–distal electrode channels in seven of the eight participants (Fig. 4, Table 1). Significantly larger ratio values occurred in distal channels compared to proximal channels in all individuals, except one (*p *⩽ 0.004, all cases). In the one participant where a significant linear relationship did not exist (Fig. 4, S2), the largest ratio values still occurred in more distal channels (channels 8 and 10).

## 4 Discussion

We wished to identify whether the maintenance of standing posture in humans and electrical stimulation of the motor nerve resulted in activation of motor units with fibres equally distributed along the proximal–distal axis of MG. The pennate fascicle architecture in MG means that activity in muscle fibres at different proximal–distal locations are represented in different channels along the linear array (Fig. 1 and Mesin et al., 2011; Vieira et al., 2011). By comparing measures from standing and electrical stimulation of the tibial nerve, we have ensured that differences in the signal amplitudes between electrode channels did not merely reflect local morphological factors. Crucially, the only variable to change between experimental conditions was the method of activating motor units. Whilst some variation is evident in the data set, and we currently only have data from a small sample (*N *= 8), the calculated ratio values (Fig. 4) reveal a striking linear relationship along proximal–distal electrode channels. These results show that, relative to units activated during electrical stimulation, those activated during standing had a strong tendency for higher representation in the distal channels. The ratio measure means that data were assessed when each electrode channel was normalised to itself (Fig. 4) and we therefore interpret that differences within each electrode channel reveal different myoelectric signal amplitudes between conditions indicating that the spatial distribution of activated fibres differed between conditions.

Across our sample, linear regression analysis of the normalised ratio data showed quite large variation in the relative differences between ARV amplitudes recorded in each experimental condition (Fig. 4, Table 1) and in several instances, distal electrode channels detected higher ARV amplitudes during stimulation than during standing (Figs. 3 and 4). Stimulation of the motor nerve likely activates larger motor units, resulting in activation of a large number of fibres and resulting in extremely large potentials travelling towards the superficial aponeurosis. These potentials will be well represented in the electrode channels located immediately above the fibre ends (Figs. 1 and 2). The volume conductor effect will however mean that the amplitude of these potentials is likely to diffuse across adjacent electrode channels. During stimulation, the diffused amplitude was significantly smaller in distal compared to proximal channels (Figs. 2 and 3). During standing however, smaller motor units would have been activated, and accordingly a smaller number of fibres, resulting in smaller action potentials being detected. Differences in the signal amplitude in the distal electrode channels are therefore not as distinct between conditions as seen in the proximal channels. Crucially however, clear variation in the patterns of ARV amplitudes across channels occurred between conditions (Fig. 3), and statistical analysis revealed that across all participants there was a significant difference in signal distribution relative to electrode channel.

In muscles with pennate fascicle architectures, the local amplitude of the differential surface myoelectric signal is associated with the number of active fibres in a highly localised region close to the detecting electrode pair (Mesin et al., 2011). This factor has two implications for the analysis and interpretation of collected signals within this study. Firstly, in regions of the muscle where fascicles run more parallel to the skin action potential propagation is apparent between electrode channels (Fig. 1a and see also Vieira et al., 2011) and as such inference of the spatial distribution of active fibres in the proximal–distal axis is not possible. To ensure this factor did not bias our results channels where signal propagation was apparent have been removed from our analysis. Secondly, the localised detection volume inherent with our techniques, and the fact that analysed signals are from electrode channels located over thicker portions of MG, limits the potential of having included signals from adjacent muscles (e.g. soleus) in our protocol (see also: Mesin et al., 2011; Vieira et al., 2011). It should however be noted that, while the methodology provides a means of recording from localised regions of the muscle, it does mean our results do not take any account of changes which may have occurred outside the longitudinal plane of the linear electrode array and therefore limits our sample of detected motor units. Alternative techniques, such as 2D electrode arrays (Vieira et al., 2010), transverse relaxation time (T2) weighted MRI (Kinugasa et al., 2011) and positron emission tomography (Pappas et al., 2001), can provide information of activation patterns across a larger muscle volume. For example, Kinugasa et al. (2011) showed non-uniform spatial distribution of activity between plantar flexion movements with different loads, with the location of highest activation moving from medial-distal region at low load (20% of maximum weight moved) to lateral-central muscle region at a higher load (60% of maximum weight moved), a shift which is in agreement with findings presented here.

Taking assessment of the validity of recorded myoelectric signals noted above, we have found a tendency for higher relative representation of postural units, not activated during stimulation, in the distal electrode channels (Figs. 3 and 4). Therefore, within the population tested here, we suggest that, relative to the distribution evoked by stimulation, the distal portion of MG makes a larger contribution to maintenance of postural stability than the proximal muscle portion. As distal fibres are physically closer to the Achilles tendon, and are less pennate than more proximal fibres (Shin et al., 2009), it is likely they have a greater mechanical advantage for generation of ankle plantar flexion torque. Recent evidence suggests a strong degree of economy within postural control mechanisms, with the musculoskeletal system tolerating a degree of postural sway during upright stance to ensure a stable posture is achieved with near-minimum muscle activation (Kiemel et al., 2011). We therefore suggest the potential for a further mechanism to facilitate low levels of activation: specifically that, fibres activated in MG are in the optimum location to effectively contribute to plantar flexion torques. As such, even within muscles considered to have a single line of action, regional variation in fascicle architecture may have functional significance. It should also be noted that the distal portion of MG is more closely associated with the underlying soleus muscle, through their joint insertion to form the Achilles tendon. As such utilising the distal portion of MG for postural control may facilitate synergistic activity with the soleus muscle and provide a further mechanism for economy within the context of postural control.

Coupled with previous work, exploring the surface representation of action potentials in muscles with pennate fascicle architecture (Mesin et al., 2011; Vieira et al., 2011), our results provide further evidence that the influence of fascicle architecture on the distribution of myoelectric signal properties at the skin surface is an important consideration for experimental design. In muscles with pennate architecture, recording from a single site is unlikely to provide myoelectric data representative of activity in the muscle as a whole. Fascicle geometry and the experimental protocol(s) to be completed should therefore inform decisions on the location and number of electrodes to be used during any experimental protocols where surface myoelectric data are to be collected.

### 4.1 Distribution of motor unit populations in MG

The previous reports of localised patterns of activity in MG (Staudenmann et al., 2009; Wakeling, 2009; Kinugasa et al., 2011) coupled with our preliminary findings that, relative to units activated during electrical stimulation, units activated during standing are more highly represented in the distal portion of MG, mean it is warranted to consider how regional variation in activation patterns may occur. Skeletal muscles are composed of a spectrum of intrinsic properties determined by the motor units they are composed of. The experimental protocol applied here is likely to have resulted in activation of motor unit populations representing different extremes of this spectrum within MG. The population activated during standing, when force requirements are low, were likely to be dominated by the smallest, most excitable units within the muscle. In contrast, electrical stimulation of the motor nerve likely leads to larger diameter nerve fibres, associated with larger motor units (McPhedran et al., 1965; Wuerker et al., 1965), being activated at lower stimulation intensities (McNeal, 1976), resulting in activation of a population of motor units dominated by larger units (Hennings et al., 2007). Our results may therefore provide preliminary evidence that fibres of different motor unit populations are spatially distributed along the proximal–distal length of MG, with faster units preferentially represented in the proximal region and slower units more highly represented in the distal region. As such, while we cannot exclude the potential for uneven synaptic input across MG motoneurons, it appears that the distribution of physiological properties along the length of MG may contribute to the preferential activation of the distal portion during standing and may also influence the regional patterns of activation reported elsewhere (Staudenmann et al., 2009; Wakeling, 2009; Kinugasa et al., 2011).

Regional distribution of fibre types have previously been reported in a range of muscles (for review see: Kernell, 1998), however such regionalisation tends to correspond to sub-division of the muscle into separate compartments, each innervated by a single primary nerve branch (Weeks and English, 1987; DeRuiter et al., 1995). In humans, MG is innervated by a single primary nerve branch (Wolf and Kim, 1997) and as such organisation of intrinsic properties within its volume is surprising. It has been shown that fascicle geometry (Narici et al., 1996; Shin et al., 2009) and compliance of the deep aponeurosis (Kubo et al., 2002) vary along the length of MG and as such distributing different motor unit types along the muscle may enable them to exploit these geometric and passive material characteristics to optimise intrinsic properties. For example, during contraction, changes in intramuscular pressure are predicted to vary greatly within individual skeletal muscles (Otten, 1987; van Leeuwen and Spoor, 1992) and may disrupt blood supply. The consequences of impeded circulation are likely to have greater impact on the fibres of smaller motor units, which rely more heavily on oxidative metabolism. Mathematical modelling, based on human MG, predicts higher intramuscular pressures in proximal muscle regions with a gradual reduction towards distal regions (van Leeuwen and Spoor, 1992). It is therefore possible that if the fibres of smaller motor units are located in the distal portion of MG they may benefit from less disruption of their blood supply compared to the fibres of motor units in more proximal regions. However, some caution is required with regard these suggestions, as the present data set showed some variability across participants (e.g. slope co-efficients in ratio data, Table 1), and our sample size is relatively small (*N *= 8). In addition, as we are likely to have only activated a very small proportion of the units at each end of the property spectrum within MG, further work is required to determine: (i) how much our results generalise to a larger population; (ii) the full extent to which different motor unit populations may be spatially organised; and (iii) the functional implications of such organisation.

## Conflicts of Interest

There are no conflicts of interest.

## Figures and Tables

**Table 1:** Results of linear regression analysis of ARV amplitudes from standing and stimulation protocols (Fig. 3) and for calculated ratio values (Fig. 4). Results where *p *> 0.05 are shown in italics with *r*^2^ values omitted.

Subject	Standing		Stimulation		Ratio	
	*r*^2^	Line equation	*r*^2^	Line equation	*r*^2^	Line equation
S1	0.91	10.30 + 0.451x	–	*69.69 − 0.14x*	0.70	0.11 + 0.08x
S2	0.45	4.74 + 0.14x	–	*78.46 − 2.59x*	–	*0.06 + 0.01x*
S3	0.45	13.32 − 0.23x	0.81	113.93 − 8.15x	0.88	−0.03 + 0.05x
S4	0.81	5.80 + 0.52x	0.73	19.81 − 1.26x	0.89	0.20 + 0.10x
S5	–	*3.08 + 0.03x*	0.87	44.63 − 4.00x	0.84	−0.14 + 0.08x
S6	–	*8.10 + 0.08x*	0.56	126.50 − 5.83x	0.54	0.06 + 0.01x
S7	0.60	14.15 + 1.33x	0.74	172.45 − 12.74x	0.84	0.03 + 0.04x
S8	–	*16.67 + 0.63x*	0.76	83.20 − 7.33x	0.76	0.02 + 0.10x
